# Oxaliplatin-induced cardiotoxicity in mice is connected to the changes in energy metabolism in the heart tissue

**DOI:** 10.1186/s40959-026-00453-7

**Published:** 2026-02-13

**Authors:** Junwei Du, Leland C. Sudlow, Kiana Shahverdi, Haiying Zhou, Megan S. Michie, Thomas H. Schindler, Joshua D. Mitchell, Shamim Mollah, Mikhail Y. Berezin

**Affiliations:** 1 Mallinckrodt Institute of Radiology, Washington University School of Medicine St. Louis, St. Louis, MO 63110 USA; 2 Institute of Materials Science & Engineering, Washington University, St. Louis, MO 63130 USA; 3 Cardio-Oncology Center of Excellence, Washington University School of Medicine, St. Louis, MO 63110 USA; 4 Department of Genetics, Washington University School of Medicine, St. Louis, MO 63110 USA

**Keywords:** Oxaliplatin, Cardiotoxicity, Energy metabolism, Glycolysis switch

## Abstract

Oxaliplatin is a platinum-based alkylating chemotherapeutic agent used for cancer treatment. At high cumulative dosage, the negative effect of oxaliplatin on the heart becomes evident and is linked to a growing number of clinical reports. The aim of this study was to determine how chronic oxaliplatin treatment causes the changes in energy-related metabolic activity in the heart that leads to cardiotoxicity and heart damage in mice. C57BL/6 male mice were treated with a human equivalent dosage of intraperitoneal oxaliplatin (0 and 10 mg/kg) once a week for eight weeks. During the treatment, mice were followed with ECG, histology and RNA sequencing of the heart. We identified that oxaliplatin induces strong changes in the heart and affects the heart’s energy-related metabolic profile. Histological post-mortem evaluation identified focal myocardial necrosis infiltrated with a small number of associated neutrophils. Accumulated doses of oxaliplatin led to significant changes in gene expression related to energy related metabolic pathways including fatty acid (FA) oxidation and glycolysis leading to the glycolysis switch. Our study can be used for the development of diagnostic methods to detect oxaliplatin-induced cardiotoxicity at an early stage and identifying therapeutic methods to minimize heart failure.

**Supplementary Information:**

The online version contains supplementary material available at 10.1186/s40959-026-00453-7.

## New & Noteworthy

This study presents the detrimental impact of chronic oxaliplatin treatment on heart metabolism in mice, linking high accumulative dosages to cardiotoxicity. This cardiotoxicity was accompanied by significant changes in gene expression related to energy metabolic pathways that led to a switch between normal fatty acid metabolism to a toxic glycolysis-based metabolism. Our findings can help to identify therapeutic methods to minimize heart failure, preventing heart damage in chemotherapy treated patients.

## Introduction

Oxaliplatin is the first line of defense against colorectal cancers and is also used in other malignancies, including gastric, pancreatic, and advanced hepatocellular carcinomas. Oxaliplatin’s efficacy is limited by its off-target toxicity leading to many acute and chronic side effects that include chemotherapy induced peripheral neuropathy, anemia, nausea, vomiting, ototoxicity, renal toxicity, and hypersensitivity [1–5] with the search of less toxic but still efficient platinum based chemotherapies remains an active area of research [6]. One of the typical, but less studied side effects of oxaliplatin is its adverse effect on the heart. Patients treated with oxaliplatin often experience rapid breathing, chest pain, increased heart rate, and an irregular heartbeat. Although emergency situations in oxaliplatin-treated patients are relatively rare compared to other drugs like anthracyclines [7, 8] or 5-fluorouracil [9] it is a rising concern given the increasing number of patients treated with oxaliplatin and other platinum based drugs [10]. A growing number of cases related to severe coronary and cardiotoxicity of oxaliplatin alone [11–13] or together with 5-fluoruracil or FOLFOX [14–17] have been reported.

Chemotherapy-induced alteration of heart metabolism occurs relatively quickly compared to other metabolic disorders: within several months, which corresponds to the duration of chemotherapy, rather than years, as in diabetes or other metabolic disorders. For instance, anthracycline-induced cardiotoxicity often manifests within the first year of treatment, with a median time to cardiotoxicity development of 3.5 months post-chemotherapy [18]. Similarly, cisplatin-based chemotherapy for testicular cancer has been shown to cause acute alterations in diastolic heart function and metabolic parameters within three months of treatment initiation [19]. The American Heart Association also acknowledges that chemotherapy-related cardiotoxicity can present acutely or within a few months of treatment, emphasizing the need for early detection and management [20].

One of the hallmarks of heart disorders associated with diabetes and other metabolic disorders is the metabolic switch from fatty acid (FA) oxidation - the primary energy source of the heart - to glycolysis. In diabetes, this metabolic remodeling leads to decreased cardiac efficiency and energetics, contributing to the progression of cardiac dysfunction in diabetic patients [21]. We envisioned that oxaliplatin affects the heart in a similar way, by reducing expression of enzymes involved in FA metabolism and activating the glycolytic pathway. Such a switch can lead to increased production of toxic byproducts such as lactate and a decrease in energy production [22].

Using ECG, post-mortem histology, and RNA-seq, we demonstrated direct damage to the heart caused by oxaliplatin in otherwise healthy mice. Genetic analysis identified a number of differentially expressed genes (DEGs) related to impaired energy-related metabolic processes and pathways. We observed that strong suppression of FA metabolism followed by the activation of glycolysis potentially leading to stressful condition. These results provide key insights into the regulation of metabolic flux during oxaliplatin treatment and suggest potential diagnostic and therapeutic targets.

## Results

### High dose oxaliplatin decreases heart rates and induces hypertrophy of the heart

**Fig. 1:**
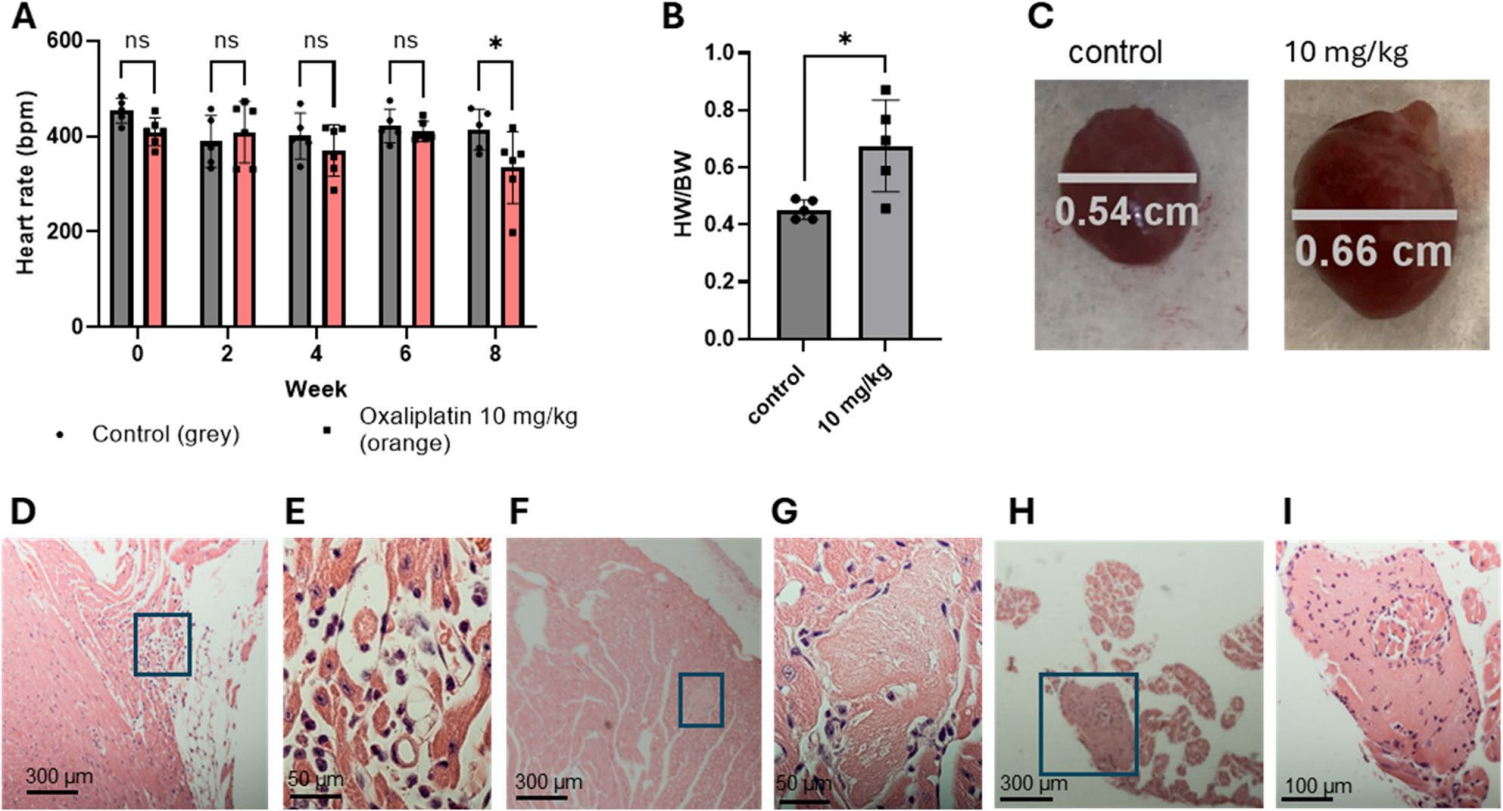
Gross effects of oxaliplatin on murine hearts. **A**. Oxaliplatin significantly slowed resting heart rates after 8 weeks of treatment. By week 8, oxaliplatin-treated mice had a significantly lower resting heart rate. * *p* < 0.05, Anova **B**: Heart weight/body weight (HW/BW, mg/g) index for mice treated with oxaliplatin. Correlation between the heart size and the Heart weight/body weight index. The Pearson correlation between the size and the HW/BW = 0.86. **C**: Example images of the enlarged heart measured at greatest width in the ventricles. **D**: Right atrial histology from a mouse treated with oxaliplatin for 8 weeks. Inset box is magnified in **E.** There is interstitial infiltration by neutrophils and mononuclear cells; **F**: Ventricular histology near the apex of the mouse heart. Insert box is magnified in **G**. Focal myocardial necrosis is evident. This is a small focus of coagulative necrosis, with loss of cross-striations and loss of nuclei; **H**: Ventricular histology from near the right atrial appendage. Inset box is magnified in **I**. Focal myocardial necrosis is evident in the right atrial appendage that also shows infiltration of a small number of associated neutrophils. *N* = 5 per group, all males

Mouse heart rates were significantly affected at high-accumulated oxaliplatin dosage. Heart rates (HR) of the anesthetized, control mice for weeks 0–8 averaged 416 ± 45 bpm well within the range of published data for isoflurane anesthetized mice [23] (Fig. 1A). The average HR of the 10 mg/kg group at week 8 was 305.5 ± 74.6 bpm. In particular, one of the oxaliplatin treated mice at week 8 showed a heart rate of 198.6 ± 2.53 bpm.

Oxaliplatin significantly affects the body weight **(BW**) of the animals (**Supplementary Information**,** Figure ****S1**) and the weight of the heart. The heart weight/body weight (HW/BW) index, whose normal value is around 0.4–0.45 mg/g for wild-type mice, showed a dose-dependent increase compared to the control mice reaching 0.8 mg/g for the most affected mice (Fig. 1B**)**. The morphology of the heart in oxaliplatin-treated mice resembled dilated cardiomyopathy that (Fig. 1C**)** has been described in small animals in various cardiovascular diseases such as hypertension, hypertrophic cardiomyopathy myocardial infarction, valvular heart disease, and heart fibrosis [24, 25].

Varying degrees of heart damage in mice treated with oxaliplatin became evident during histological examination. Small areas of the heart showed inflammation associated with neutrophil and mononuclear cell infiltration (Figs. 1D **– I**, for more images demonstrating the damage to the heart are given in **Supplemental Information**,** Figures ****S3****-S6).** Regions of necrosis with loss of striation, necrotic coagulation, and inflammation associated with the infiltration of a small number of inflammatory cells such as neutrophils can also be seen.

## RNA-seq demonstrates that heart metabolism is strongly affected by oxaliplatin

**Fig. 2:**
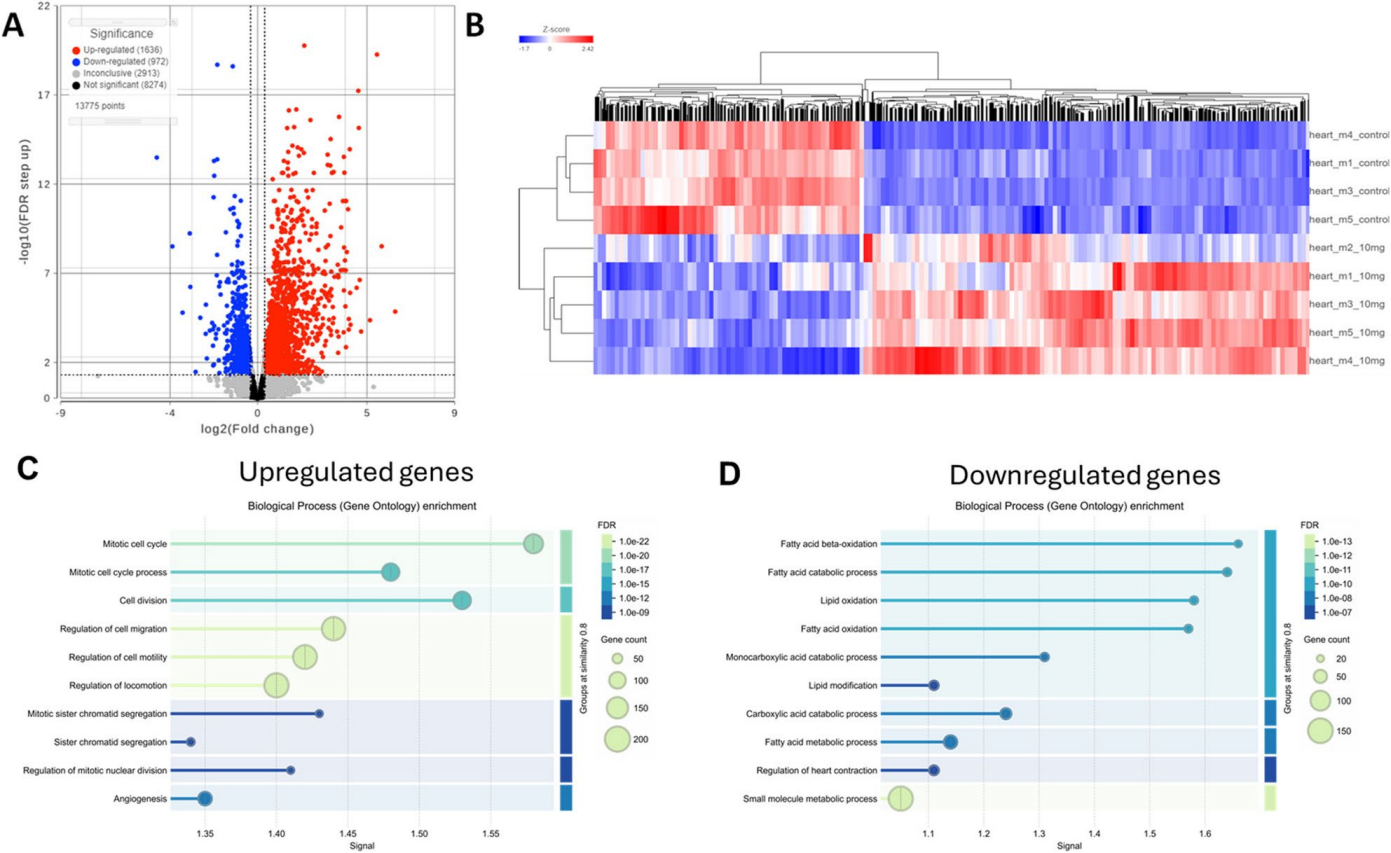
Effect of oxaliplatin on the RNA transcription in the heart. **A**: Volcano plot representation: threshold (FC) -1.25 and 1.25, threshold FDR < 0.05. Total number of identified genes in the heart = 13,755, upregulated = 1,636, downregulated = 972 (~ 19% genes affected). **B**: Heatmap of these DEGs: control dextrose-treated mice – top 4 rows, oxaliplatin -treated (bottom 5 rows). **C**: Top 10 processes affected by oxaliplatin by GO pathway enrichment analysis of upregulated DEGs indicates mitotic cell cycle and cell division processes as top affected pathways. **D**: Top 10 processes of downregulated DEGs point to fatty acid metabolism as the top affected pathways. The “Signal” metric represents score that integrates enrichment significance and effect size. Higher values indicate greater statistical significance of enrichment. RNA-seq analysis was performed on *n* = 4 control and *n* = 5 oxaliplatin-treated mice, following exclusion of one control sample due to low RNA quality

From 13,775 identified by RNA-seq in the heart, 2608 genes, or ~ 19%, were differentially expressed (FC -1.25 to 1.25, FDR < 0.05) (Fig. 2A). Heatmaps for the gene expression of all DEGs in the heart tissue combined with the hierarchical clustering analysis demonstrated an excellent separation between the oxaliplatin treated and control groups (Fig. 2B**)**. From the GO Biological Processes analysis (STRINGdb) [26], we found significant enrichments of mitotic and metabolic processes as shown in Figs. 2C-D.

Since mature cardiomyocytes are largely post-mitotic, the upregulation of mitotic cell cycle pathways (Fig. 2C**)** in the hearts of oxaliplatin-treated mice suggests a stress-induced response. DNA damage and oxidative stress from oxaliplatin may trigger a cell cycle re-entry attempt in cardiomyocytes, likely through G2/M checkpoint activation [27] (e.g., *Trp53* (FC = 1.56), *Cdc25c* (FC = 17.21)), even if these cells do not complete division. However, it is also plausible that the observed upregulation of mitotic pathways may stem from cardiac fibroblasts. Cardiac fibroblasts are known to proliferate in response to fibrosis and angiogenesis, and the elevation of fibrotic markers such as *Mmp2*,* Fn1*,* and Fstl1* (see above) supports this hypothesis [28].

Given the strong impact of oxaliplatin on FA metabolism (Fig. 2D**)**, we focused on energy related genes. KEGG identifies 1340 genes relevant to the entire metabolism [29]. From this pool we narrowed our focus to 75 genes directly involved in the major energy-related pathways including FA transport and oxidation, glycolysis, TCA cycle and others. The full list of these genes and pathways is given in **Table ****S2** and detailed below.

We conducted qPCR for a few selected genes to validate RNA-seq data. Those genes were selected due to their established involvement in cardiovascular diseases including heart failure and atherosclerosis. Similar to RNA-seq, qPCR from the heart tissues showed elevated expression of heart-related disease markers including *Nppb* (b-type natriuretic peptide, a cardiac hypertrophic marker), *Cdk1*, (cyclin-dependent kinase 1, key cell cycle regulator), and *Lgals3* (galectin-3). Elevated expression of genes such as *Nppb*, *Cdk1*, and Lgals3 indicate cardiac damage and inflammation, and their expression is known to be upregulated in response to cardiotoxic agents and hemodynamic stress (**Figure ****S1**) [30–33]. *Lgals3* has been proposed to be a marker for detecting cardiotoxicity in cancer patients undergoing chemotherapy [31, 34]. Other markers of heart damage include *Aebp1* (FC = 2.07) that is elevated in patients with cardiac fibrosis and hypertrophic cardiomyopathy [35] and others elevated fibrosis related genes *Mmp2* (FC = 2.30), *Fn1* (FC = 4.29), *Fstl1* (FC = 4.06), see **Supplementary Information**,** Table ****S1**** – all DEGs**).

## Oxaliplatin disrupts fatty acid utilization in cardiomyocytes

Transcriptomic analysis revealed a marked dysregulation of fatty acid handling pathways. Genes involved in fatty acid transport from the capillary lumen to the endothelium were significantly upregulated, whereas key enzymes responsible for mitochondrial fatty acid β-oxidation were broadly downregulated.

**Fig. 3:**
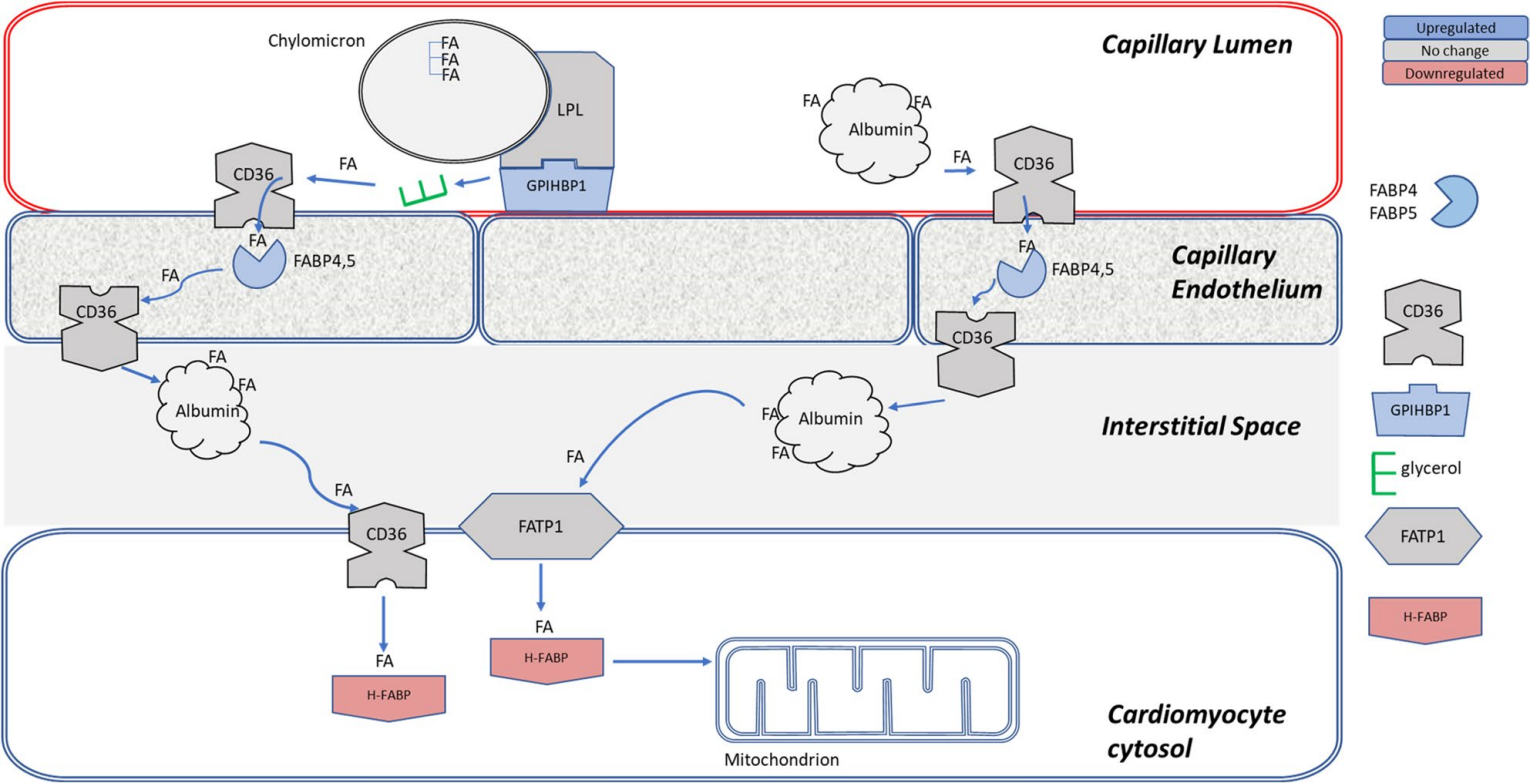
**FA transport from capillary lumen to endothelium is upregulated by oxaliplatin.** Abbreviations: Lipoprotein lipase, LPL (*Lpl*), glycosylphosphatidylinositol-anchored protein 1 GPIHBP1 (*Gpihbp1).* CD36 FA transporter (*Cd36*). FA binding proteins 4 and 5, FABP4,5 (*Fabp4* and *Fabp5*), FA translocase transport protein FATP1 (*Slc27a1)*, heart FA binding protein 3, H-Fabp (*Fabp3*). Based on RNA-seq analysis on *n* = 4 control and *n* = 5 oxaliplatin-treated mice

Cardiac FAs are catabolized from triglycerides (TGs) from triglyceride-rich lipoproteins (chylomicrons) or via albumin-bound FAs in plasma [36] as shown schematically in Fig. 3. Delivery of FAs to cardiomyocytes begins with FA transport from capillaries to the endothelium, where TGs are hydrolyzed by lipoprotein lipases (LPL) with the help of high-density lipoprotein-binding protein 1 Gpihbp1 [37], encoded by overexpressed *Gpihbp1* (FC = 2.51) (**Figure S7**). Elevated *Gpihbp1* expression has been previously observed in fasting mice [38]. Free FAs are imported by CD36 [39]. Once in the capillary endothelial cytoplasm, FAs are transported to abluminal epithelial membrane by FA binding proteins FABP4 and FABP5, encoded by *Fabp4* and *Fabp5* upregulated in the oxaliplatin group [40]. The overexpression of these genes strongly and positively correlates with *Gpihbp1* expression (*Pearson correlation values* (*Fabp4* vs. *Gpihbp1) P =* 0.944, *P (Fabp5* vs. *Gpihbp1) P =* 0.984, **Figure S4**) suggesting highly coordinated process for the hydrolysis of TGs and the transport of liberated FAs through the capillary endothelial. FAs are then taken up by cardiomyocytes by CD36 or by FATP1 that translocate FAs across the sarcolemma into the cardiomyocyte cytoplasm [39, 41] where they are transported by cytoplasmic heart-type fatty acid-binding protein to mitochondria (H-FABP, *Fabp3*). Overall, the upregulation of the key FA processing and FA binding proteins suggest a strong attempt to increase the delivery of the FA to cardiomyocytes.

**Fig. 4:**
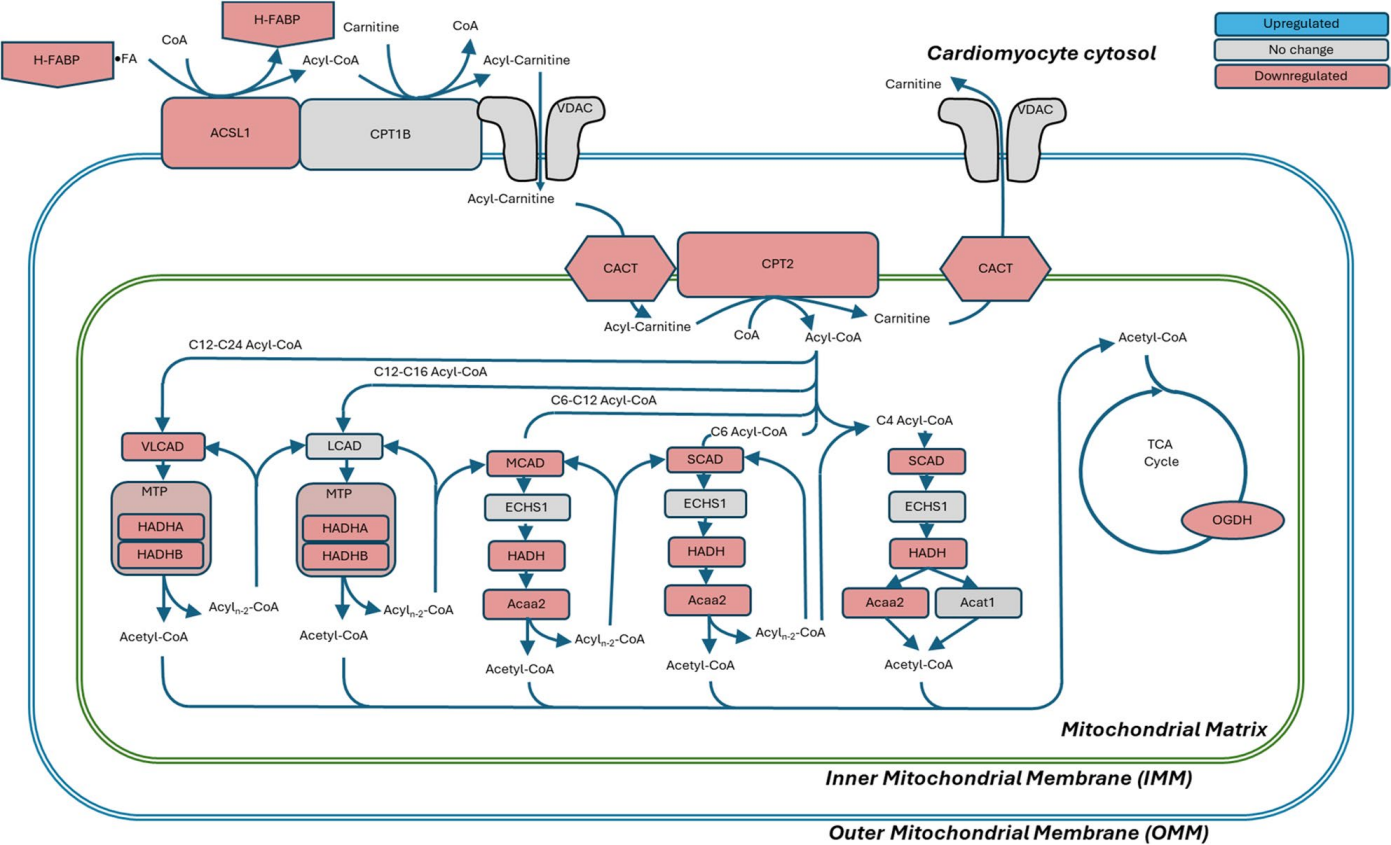
**Fatty acids ß-oxidation pathways in cardiomyocytes mitochondria are strongly downregulated by oxaliplatin.** Abbreviations: ACAA2, Acetyl-CoA Acyltransferase 2 (Acaa2), ACAT1, acetyl-CoA acetyltransferase 1 (*Acat1)*, ACSL1, acyl-CoA synthetase long chain family member 1 (*Acsl1*); CACT, Carnitine acylcarnitine translocase (*Slc25a20*); CPT1B, Carnitine O-palmitoyltransferase 1 (*Cpt1b*); CPT2, Carnitine O-palmitoyltransferase 2 (*Cpt2*); ECHS1, enoyl-CoA hydratase, short chain 1 (*Echs1*); HADH, hydroxyacyl-CoA dehydrogenase (*Hadh*); HADHA and HADHB, hydroxyacyl-CoA dehydrogenase alpha and beta subunits of the mitochondrial trifunction protein complex (*Hadha* and *Hadhb*); H-FABP, heart fatty acid binding protein (*Fabp3*); LCAD, long chain acyl-CoA dehydrogenases (*Acadl*); MCAD, medium chain acyl-CoA dehydrogenase (*Acadm*); MTP, mitochondrial trifunction protein complex; OGDH, oxoglutarate dehydrogenase (Ogdh); SCAD, small chain acyl-CoA dehydrogenase (*Acads*); VLCAD, very long chain acyl-CoA dehydrogenases (*Acadvl*); VDAC, voltage dependent ion channels (*Vdac1*, *Vdac2*, or *Vdac3*). The pathway is derived from KEGG pathway map00071. No upregulated genes were detected. Based on RNA-seq analysis on *n* = 4 control and *n* = 5 oxaliplatin-treated mice

Within cardiomyocytes, FAs bind to H-FABP and are transported to mitochondria for β-oxidation and energy production [42]. Lower *Fabp3* expression (FC = − 1.44) (**Figure S7**) suggests impaired FAs transport to mitochondria. Formed FAs − H-FABP aggregates are transported to the outer mitochondrial membrane (OMM) (Fig. 4). Because FAs cannot penetrate the OMM, FAs must first undergo esterification into acyl-CoA [43]. This reaction is carried out by OMM-bound long chain acyl-CoA synthetase encoded by *Acsl1* [44, 45] along with several other synthetases for different FA chain lengths. Downregulation of *Acsl1* (FC = − 1.3) may indicate a lower conversion rate and less availability of the substrate for the mitochondria. A high correlation between the expression of *Fabp3* and *Acsl1* (*P* = 0.951) suggests tight coordination between the transport of the cytosolic FA toward the mitochondria membrane and its conversion into acyl-CoA (**Figure S8**).

The formed acyl-CoA is converted to acyl-carnitine by carnitine palmitoyltransferase 1B (*Cpt1b*) and diffuses into the mitochondrial intermembrane space via voltage dependent anion channels (Vdac1-3) (Fig. 4). From here, acyl-carnitines are transported through the inner mitochondrial membrane (IMM) by translocase CACT (encoded by *Slc25a20*) [46]. Lower expression of *Slc25a20* (FC = − 1.43) (**Figure S9**) results in the reduced availability of FAs for mitochondrial β-oxaliation. Once the acyl-carnitines reach the mitochondrial matrix, the FA chain is re-transferred to CoA by carnitine palmitoyltransferase 2, (*Cpt2*) [47]. Predictably, *Cpt2* was also downregulated (FC = − 1.7) (**Figure S9**). Given that this enzyme controls a rate limiting step in the β-oxidation pathway [48], the entire process of downstream β-oxidation would likely be substantially reduced.

β-Oxidation of FAs into acetyl-CoA fragments supporting ATP synthesis is the final portion of FA metabolism. This process is significantly affected by oxaliplatin with many enzymes for saturated and non-saturated FA β-oxidation being downregulated as illustrated in Fig. 4. For saturated FAs, three out of four dehydrogenase enzymes that perform the first step producing a corresponding enoyl-CoA were downregulated: SCAD (*Acads*, FC = − 1.47), MCAD (*Acadm*, FC = − 1.43), and VLCAD (*Acadvl*, FC = − 1.31) (**Figure S10**). Other downregulated enzymes on this pathway include *Hadh*, (FC = − 1.5) and both subunits of the mitochondrial trifunctional protein (MTP) encoded by *Hadha* (FC = − 1.53) and *Hadhb* (FC *= −* 1.47). These subunits perform multiple steps in β-oxidation and are essential for the normal functioning of the heart [49, 50]. Similarly, β-oxidation of unsaturated FAs utilizes a downregulated set of enzymes in oxaliplatin treated mice including *Eci1* (FC = − 1.49), *Decr1* (FC = − 1.44), and *Ech1* (FC = − 1.78) (**Figure S11**). Downregulation of multiple enzymes in the β-oxidation pathway suggest the heart would be deprived of a significant source of energy to synthesize ATP. Since cardiomyocytes are unable to take up adequate levels of FAs, the heart must utilize other sources of energy such as switch to glycolysis.

## Oxaliplatin induces a transcriptional shift toward glycolytic metabolism

Suppression of FA metabolism was accompanied by transcriptional upregulation of glycolytic pathways. Notably, the expression of the energy-sensing PGC1α gene was increased (*Ppargc1a*, FC = 1.37, **Figure S12**). This protein enables the heart to respond to many energy metabolism-related stimuli including cold, fasting, exercise, and changes in substrate availability [51]. The cardiac-specific overexpression of PGC1α in mouse models has been often associated with dilated cardiomyopathy [52] and accelerated cardiac aging [53].

**Fig. 5:**
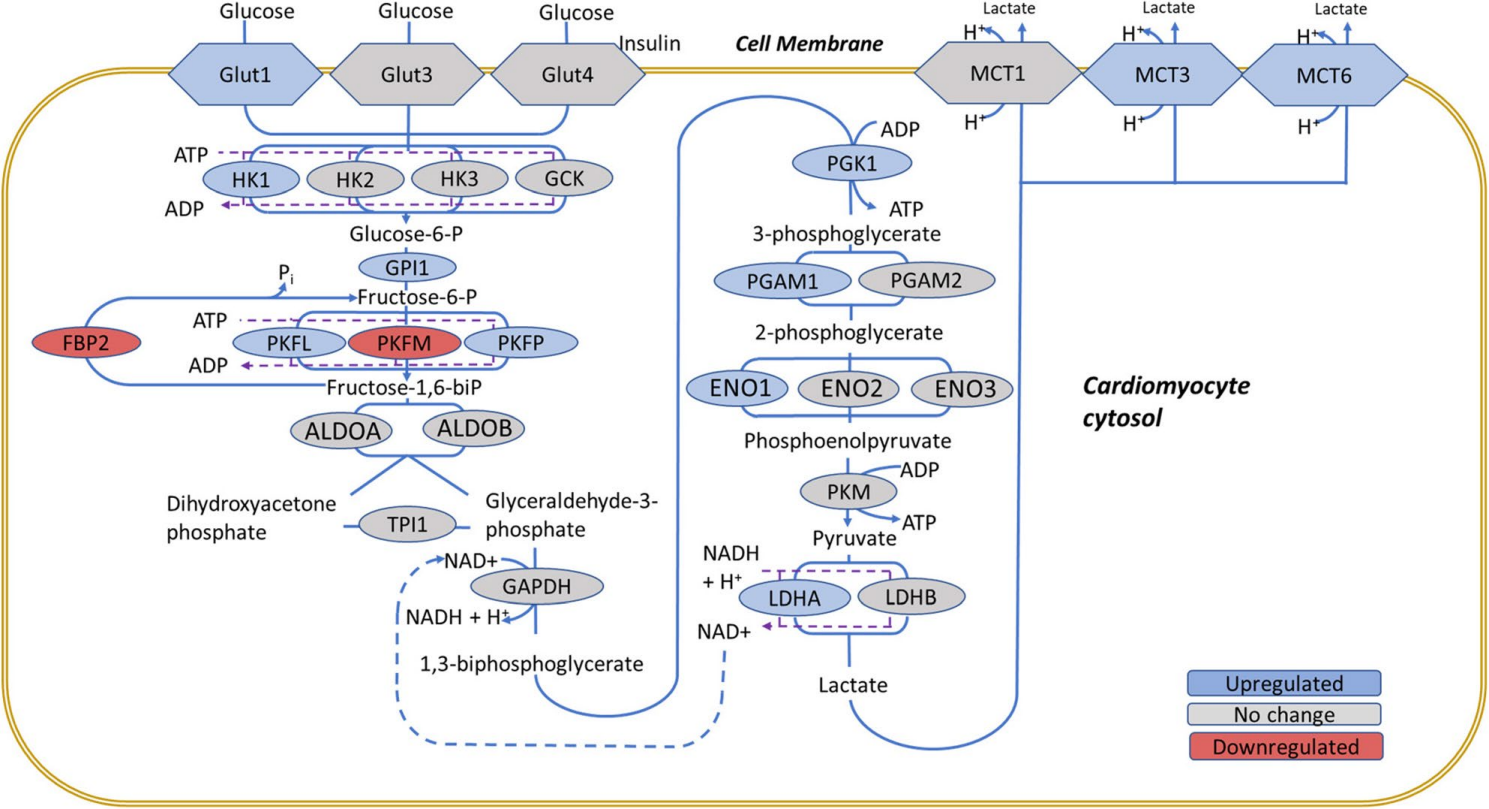
Glycolysis in cardiomyocytes is upregulated in the oxaliplatin treated mice. Abbreviations: ALDOA, aldolase A (*Aldoa*); ALDOB, aldolase B (*Aldob*); ENO1/2/3, enolase 1, 2 and 3 (*Eno1*,* Eno2* and *Eno3*); FBP2, fructose-bisphosphatase 2 (*Fbp2*), GAPDH, glyceraldehyde-3-phosphate dehydrogenase (*Gapdh*); GCK, glucokinase (*Gck*); GLUT1, glucose transporter 1 (*Slc2a1*); GLUT3, glucose transporter 3 (*Slc2a3*); GLUT4, Insulin-responsive glucose transporter 4 (*Slc2a4*); GPI1, glucose-6-phosphate Isomerase 1 (*Gpi1*); HK1, HK2, HK3 hexokinase 1, 2 ,3 (*HK1*,* HK2*,* Hk3*); LDHA, LDHB, lactose dehydrogenase A, B *(Ldh1*,*Ldhb*); MCT1,MCT3,MCT6 monocarboxylic acid transporters 1, 3, 6 (*Slc16a1*,* Slc16a3*,* Slc16a6*); PGAM1, phosphoglycerate mutase 1 (*Pgam1*); PGAM2, phosphoglycerate mutase 2 (*Pgam2*); PGK1, phosphoglycerate Kinase 1, (*Pgk1*); PFKL, phosphofructokinase-liver type (*Pfkl*); PKFM, phosphofructokinase-muscle type (*Pfkm*); and PKFP, phosphofructokinase-platelet type (*Pfkp*); PKM, pyruvate kinase-muscle type (*Pkm*); TPI1, topoisomerase 1 (*Tpi1*). The schematics is in part based on KEGG Pathways and ref [55]. Based on RNA-seq analysis on *n* = 4 control and *n* = 5 oxaliplatin-treated mice

Not surprisingly, *Ppargc1a* expression showed strong correlations with other upregulated genes in the glycolysis pathway, such as enolase 1 (*Eno1*,* P* = 0.94), glucose-6-phosphate isomerase 1 (*Gpi1*,* P* = 0.93), and phosphofructokinase, platelet *(Pfkp*,* P* = 0.93) (**Figure S13)**. This confirms that PGC1α facilitates a metabolic switch to glycolysis in the oxaliplatin treated group. This switch was further corroborated by the upregulation of many other genes in the glycolysis pathway as illustrated in Fig. 5 and tabulated in **Table ****S2****.** Among them is a glucose transporter GLUT1 encoded by *Slc2a1* (**Figure S12**) which facilitates glucose uptake in cardiomyocytes. While GLUT1 is essential during heart development, its role declines postnatally as GLUT4 becomes dominant. Under chronic stress, however, GLUT1 is re-expressed in the heart as an adaptive response [54]. Other noticeable upregulated genes in the glycolytic pathway include *Hk1* (FC = 1.43), *Gpi1* (FC = 1.27), *Pfkp* (FC = 2.2), *Pgk1* (FC = 1.27), *Eno1* (FC = 1.74), *Pgam1* (FC = 1.45), *Ldha* (*F*C = 2.08) (**Figure S14**).

Altogether, the transcriptional upregulation of glycolysis reflects maladaptive compensation for ATP generation via lactic acid production. As a result of this compensation the difference between the levels of ATP measured in the hearts between the oxaliplatin and control group did not show a statistical significance **(Figure S15).**

## Discussion

Unlike other organs that rely heavily on glucose for energy, the healthy heart in humans and rodents relies predominantly on FAs. The healthy mouse heart utilizes 70% FAs and less than 30% carbohydrates as energy sources for ATP production [56]. Small contributions also come from branched chain amino acid catabolism and ketone bodies. Oxaliplatin induced a strong metabolic stress on the heart, starving the mitochondria from the required level of FAs to produce sufficient ATP. Despite the increased transport of the FA to cardiomyocytes the heart experienced strong FA deficiency. However, the heart is highly adaptable and can easily switch from one source of energy to another when required, such as from FA to glycolysis. This process known as the “glycolysis switch”, is common in mammals and is observed in the heart under stress conditions.

The cumulative oxaliplatin dose appears to be the most significant risk factor for the development of oxaliplatin cardiotoxicity. No significant changes in the cardiac activity of mice by ECG receiving a human-equivalent dose of oxaliplatin was observed in the first 4 weeks of the treatment. However, after 8 weeks, the heart rates were significantly decreased from a normal 370–460 bpm range [23] to 200–370 bpm at the high-accumulated dosage, while the size of the heart became larger. At this time point, the histology of the heart also revealed a number of lesions evidencing moderate to severe necrosis, fibrosis and inflammatory cell infiltration.

The observed energy-related metabolic changes were tightly connected with other processes affected by oxaliplatin. One of the well-established effects of oxaliplatin is a strong hematological toxicity that decreases red blood cells [57]. The reduced level of red blood cells leads to lower oxygen concentration delivered to the cardiac tissue. In that regard the change from FA oxidation to glycolysis could be beneficial since anaerobic glucose metabolism is more ‘oxygen sparing’.

Another potential driver for switching from FA to glycolysis is an extensive loss of fatty tissue caused by oxaliplatin. Oxaliplatin is known to induce fat and muscle loss [58, 59]. The loss of adipose tissue decreases the availability of FAs for oxidation, necessitating a metabolic shift towards glycolysis.

While this glycolysis switch initially provides the heart with increased energy production, sustained activation of glycolysis and chronic reliance on glucose metabolism can cause metabolic dysfunction and contribute to heart failure. The heart is not well adapted to rely solely on glucose metabolism and is not able to efficiently utilize glucose for energy production in the long term. Chronic glycolysis leads to the accumulation of toxic intermediates and oxidative stress, and the upregulation of glycolytic enzymes can lead to altered calcium handling and impaired contractile function of the heart. These processes can result in remodeling in the heart, leading to hypertrophy, inflammation, and fibrosis. Indeed, RNA-seq showed changed expression levels of known markers of the heart damage *Nppb*,* Myh6*,* Col3a1*, and *Mmp2* [60–63]. The prevention of the loss of fatty acid metabolism can potentially reverse the switch ultimately improving the efficacy of oxaliplatin-based chemotherapy with no or minimum heart damage.

### Limitations and future directions

This pilot study, conducted with a limited number of animals, was designed to establish a foundational understanding of oxaliplatin-induced cardiotoxicity in a murine model. While the data demonstrate cardiac involvement from multiple complementary approaches, several limitations should be acknowledged and will be addressed in future studies.

Although the metabolic signatures identified here provide mechanistic insight into oxaliplatin-induced cardiotoxicity, additional translational (protein level) and functional (i.e. echocardiography) studies will be required to determine their diagnostic or prognostic utility in patients. Oxaliplatin treatment resulted in a reduction in body weight, which may influence heart weight–to–body weight normalization and potentially overestimate cardiac hypertrophy. Accordingly, alternative normalization strategies, such as heart weight–to–tibia length ratios [64], should be incorporated in future studies.

Furthermore, the present work primarily focuses on transcriptomic changes. Future studies will include targeted protein-level validation of key metabolic enzymes and pathways to identify robust circulating and tissue-based biomarkers of oxaliplatin-induced cardiotoxicity and to further enhance translational relevance. Finally, cardiotoxic effects are expected to be both age-dependent (pediatric versus adult) and agent-specific [65, 66]. Future work will therefore focus on systematic evaluation of sex-dependent effects across different age groups in mice treated with additional platinum-based chemotherapeutic agents.

## Summary

In summary, our study demonstrates that chronic oxaliplatin exposure in mice induces significant cardiotoxicity through a marked alteration in cardiac energy metabolism. The observed shift from fatty acid oxidation to glycolysis, coupled with histological and electrophysiological evidence of cardiac damage, provides a clear mechanistic insight into oxaliplatin-induced myocardial injury. These findings are highly relevant to clinical practice: similar metabolic disturbances and cardiotoxic events have been reported in patients undergoing oxaliplatin-based chemotherapy. The identification of key metabolic markers will not only enhance our understanding of the cardiotoxic mechanisms but also suggests potential approaches for early diagnosis and therapeutic intervention. Using early biomarker screening protocols and imaging procedures (future studies), we may improve cardiac monitoring and ultimately minimize serious side effects during cancer treatment and long after. Preventing the glycolysis switch in patients undergoing chemotherapy, either with the diet or pharmacologically, might significantly improve morbidity and mortality. Given the increasing burden of both heart failure and cancer in the aging population, the development of new biomarkers, imaging methods and cardioprotective strategies will be essential to minimize the impact of cancer therapy–associated cardiac toxicity.

## Materials and methods

### Animal models

All animal studies were conducted in compliance with the Washington University Institutional Animal Studies Committee (Animal Welfare Assurance #A-3381-01) and NIH guidelines. Due to the pilot nature of this study and the logistics associated with RNA seq, transcriptomic analyses were conducted exclusively in male mice. C57BL/6 male mice 8–10 weeks old were purchased from Charles River and housed in a central animal care facility with food and water *ad libitum*. For RNA-seq analysis, five animals per group were initially processed; however, one control sample was excluded due to insufficient RNA quality, resulting in final group sizes of *n* = 4 controls and *n* = 5 oxaliplatin-treated mice. Prior to the injection, clinical grade oxaliplatin (Sandoz) was diluted in 5% dextrose. Mice were weighed and an appropriate oxaliplatin dose was delivered for that animal’s mass. The treatments consisted of control (5% dextrose), and oxaliplatin dose (10 mg/kg, accumulated dose 0–80 mg/kg). The calculation of the dosage and its relevance to the human dosage is given in **Supplemental Information**, “Calculation of the human equivalent dosage with oxaliplatin in mice”. To avoid circadian effects the mice were treated at the same time of day at 2–3 pm. At the end of the experiments, usually within 7–8 days after the last injection, mice were anesthetized via isoflurane induction (2% at 1.5 L O_2_/min) and euthanized by cervical dislocation. For electrophysiological analysis, mice were anesthetized via isoflurane induction (2% at 1.5 L O_2_/min).

Other methods including histological analysis, analysis RT-qPCR, RT-qPCR, RNA sequencing of heart and the muscle, mass-spectrometry for ATP measurement and Pearson correlation analysis are provided in the Supplemental Information Section.

## Supplementary Information

Supplementary Material 1.

Supplementary Material 2.

Supplementary Material 3.

## Data Availability

The results of RNA-seq are uploaded in the Gene Expression Omnibus (GEO) GSE233805 and publicly available from [https://www.ncbi.nlm.nih.gov/geo/query/acc.cgi? acc=GSE233805](https:/www.ncbi.nlm.nih.gov/geo/query/acc.cgi? acc=GSE233805) . MATLAB custom GUI that can be downloaded from a Github depository: [https://github.com/MikhailBerezin/IVCCA](https:/github.com/MikhailBerezin/IVCCA).

## Abbreviations

ETC: Electron transport chain
OMM: Outer mitochondrial membrane
IMM: Inner mitochondrial membrane
DEGs: Differentially expressed genes
DCM: Dilated cardiomyopathy
ICM: Ischemic cardiomyopathy
FAs: Fatty acids
