# Supplementary material for: Oxaliplatin-induced cardiotoxicity in mice is connected to the changes in energy metabolism in the heart tissue
Source: Cardiooncology. 2026 Feb 13;12:39. doi: 10.1186/s40959-026-00453-7 (PMC13005559; doi:10.1186/s40959-026-00453-7)
Supplement: Supplementary file 1 — Supplementary Material 1. [file 40959_2026_453_MOESM1_ESM.docx]

**SUPPLEMENTAL INFORMATION**

**Title:** Oxaliplatin-induced cardiotoxicity in mice is connected to the changes in energy metabolism in the heart tissue

Junwei Du, PhD^a,b^,* Leland C. Sudlow^a^, PhD*, Kiana Shahverdi, MS^a^, Haiying Zhou, PhD^a^, Megan Michie^a^, Thomas H. Schindler, MD, PhD, Joshua D. Mitchell, MD, MSCI ^c^, Shamim Mollah, PhD, Mikhail Y. Berezin, PhD ^a,b^

**Institutional Affiliations**: ^a^Mallinckrodt Institute of Radiology, Washington University School of Medicine St. Louis, MO 63110, USA; ^b^Institute of Materials Science & Engineering Washington University, St. Louis, MO 63130, USA; ^c^Cardio-Oncology Center of Excellence, Washington University School of Medicine, St. Louis, MO 63110

**Calculation of the human equivalent dosage with oxaliplatin in mice**

Oxaliplatin dosing for humans is based on a mg/surface area (m^2^) basis to account for differences in patient heights and masses. The recommended and the most used dose for oxaliplatin in metastatic colorectal cancer in human is 85 mg/m^2^ intravenously repeated every two weeks until disease progression or unacceptable toxicity (1). To convert this biweekly dosage to the weekly administration of the oxaliplatin in mice using mg/kg units we first converted the human dosage to mg/kg. The calculation for human surface area (SA) used the following equation **Eq. 1** (2):

| *SA (m^2^) = 0.20247 x height (m)^0.725^ x weight (kg)^0.425^* | (**Eq. 1**) |
| --- | --- |

For a typical human adult with height ranging from 1.52 m to 2.1 m and the body mass from 59 kg to 115 kg, the SA values range from a low of 1.56 m^2^ to a high of 2.6 m^2^. The dose equivalent range in mg/kg was then calculated from the equation **Eq. 2**:

| *Dose (mg/kg) = 85 mg / m^2^ * surface area (m^2^) ÷ mass (kg)* | **(Eq. 2**) |
| --- | --- |
|  |  |

The resulting calculated mg/kg biweekly doses ranged from 2.24 mg/kg (for a human 59 kg, 1.52 m high) to 1.54 mg/kg (for a human 113 kg, 2.11 m high) with a median of 1.79 mg/kg. Having converted the human biweekly dose from mg/m^2^ to mg/kg, we then converted the human dosage to a mouse equivalent dosage using the formula suggested by the US Food and Drug Administration (FDA) (3) (**Eq. 3**)

| *Mouse biweekly total dose (mg/kg) = Human biweekly total dose (mg/kg) * k_m_ (human) ÷ k_m_ (mouse)* | (**Eq. 3**) |
| --- | --- |

where *k_m_ (mouse)* = 3 kg/m^2^ for a hypothetical mouse of 0.02 kg, and *k_m_ (human)* = 37 mg/m^2^ for a hypothetical human. The 1.79 mg/kg human biweekly dose was equivalent to a 22.1 mg/kg mouse biweekly dose of oxaliplatin. This dose of 22.1 mg/kg exceeds the tolerance of the mouse to oxaliplatin resulting in fatalities in mice treated with doses of oxaliplatin exceeding 15 mg/kg. Therefore, mice were administered 10 mg/kg of oxaliplatin on a weekly basis.

# Methods

**Histological analysis**

At the completion of week 8, the hearts of the euthanized mice were dissected, and the masses were recorded. Hearts were fixed in 10% neutral buffered formalin. Tissues were trimmed and processed according to standard protocols, embedded in paraffin, and sectioned at 5 μm. Sections were stained via hematoxylin-eosin and further imaged on an Olympus microscope (BX51).

**RT-qPCR**

Total RNA (200 ng) was reverse transcribed using SuperScript IV VILO Master Mix (ThermoFisher Scientific) according to manufacturer’s instructions. RT-qPCR was performed with Power SYBRGreen Master Mix and QuantStudio 3 Real-Time PCR system (Applied Biosystem). Ribosomal 18S was used as an internal control and relative quantification was calculated by 2−ΔΔCt method. Primer sequences for 18S, Nppb, Cdk1, and Lgals3 are as follow: 18S forward AAGTTCCAGCACATTTTGCGAGTA, reverse TTGGTGAGGTCGATGTCTGCTTTC; Nppb forward CTTCCTACAACAACTTCAGTGC, reverse AGGTGACACATATCTCAAGCTG; Cdk1 forward AAGTGTGGCCAGAAGTCGAG, reverse TGAGAGCAAATCCAAGCCGT; Lgals3 forward AGTTATTGTCCTGCTTCGTGT, reverse GTGAAACCCAACGCAAACAG; Myh6 forward GTTAAGGCCAAGGTCGTGTC, reverse GCCATGTCCTCGATCTTGTC.

**RNA sequencing of heart and the muscle**

Hearts from euthanized animals were quickly collected and stored in ice-cold RNA*later* solution (ThermoFisher Scientific). RNA was extracted with the magnetic bead based MaxwellRSC 48 instrument using the Maxwell RSC simply RNA Tissue kit, and RNA quality was assessed using Agilent TapeStation 4200 System. From the group of 10 mice used for RNA sequencing only samples used for RNA sequencing with an integrity number (RIN) greater than 8 were used. The sample with the low RIN was discarded.

A cDNA library was prepared according to manufacturer’s protocol with Clontech SMARTer method, indexed, pooled, and sequenced on an Illumina NovaSeq 6000. RNA-seq reads were aligned to the Ensembl release 76 primary assembly with STAR version 2.5.1a. Gene counts were derived from the number of uniquely aligned unambiguous reads in the Partek Flow software package (Partek Inc.). Data were then normalized using median ratio and DEGs were identified with DESeq2 package implemented in Partek Flow. GO analysis was also performed using Partek Flow.

**Mass-spectrometry for ATP measurement**

Heart tissue was stored in -80 °C before use. ATP was extracted from mice hearts by homogenizing frozen tissue in ice-cold 50% MeOH (10 μL per mg tissue). The homogenates were centrifuged, and supernatants were collected, followed by the addition of chloroform to purify metabolites. After centrifugation, the aqueous phase of the chloroform solution was lyophilized and stored at −20°C until analysis. LC-MS was performed as described previously (62) with HPLC (1290, Agilent Technologies). Briefly, serial dilutions of ATP standards in 5 mM ammonium formate were used for calibration. The highest standard concentration of ATP used was 25 µM. ATP was detected with a Triple Quad mass spectrometer (6470 MassHunter; Agilent Technologies) under positive ESI multiple reaction monitoring. The amount of ATP was quantified by the MassHunter quantitative analysis tool (Agilent Technologies) with standard curves and concentration was normalized to weights of tissue used for ATP extraction.

## Electrocardiogram (ECG)

ECG analysis on each mouse was conducted biweekly to examine the heartbeats. ECGs were measured one week prior to the start of the experiment and 48 h after drug deliveries. Prior to ECG, mice were anesthetized via isoflurane induction (2% at 1.5 L O_2_/min). Each mouse was transferred to a battery-powered heating pad (Kent Scientific) in a grounded Faraday cage and maintained on isoflurane for the duration of the recording. Stainless steel electrodes (Fine Science Tools) were used transdermally to record the electrocardiogram. Electrodes, skin, and fur over the electrode insertion site were sanitized with isopropanol. A three-lead electrocardiogram configuration, equivalent to human V1 chest lead configuration was used (**Figure S12**). The positive electrode was inserted through the skin of the ventral chest 4 mm rostrad to the xiphoid process along the sternum, the negative electrode was inserted through the skin of the upper right chest over the pectoralis muscle. The ground electrode was inserted through the skin on the medial surface of the right thigh. An extracellular amplifier (DAM80, World Precision Instruments) was used to record the mouse ECG (gain 100x, hi pass filter 0.1 Hz, low pass filter 1 Hz). Data were collected via Powerlab 8/35 (AD Instruments) and analyzed via software (LabChart, AD Instruments).


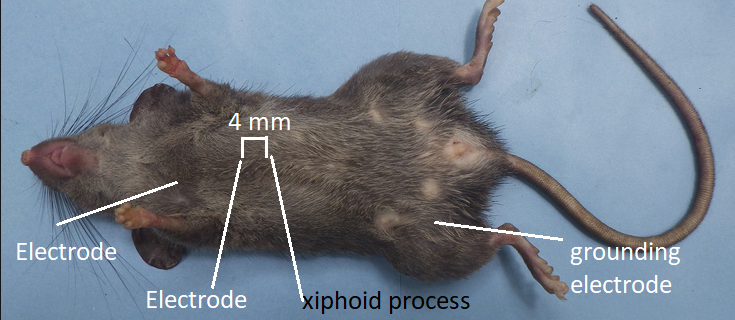


Position of ECG electrodes on isoflurane-anesthetized mouse. Skin at the insertion site and electrodes were all sanitized with isopropyl alcohol. ECG electrodes were positioned subdermal 4 mm anterior to the xiphoid process and in the upper right chest. Grounding electrode was positioned subdermal to the skin of the right thigh.

**Correlation analysis**

Correlation analysis was performed using MATLAB custom GUI that can be downloaded from a GITHUB depository: <https://github.com/MikhailBerezin/IVCCA>.

**SUPPLEMENTARY FIGURES**


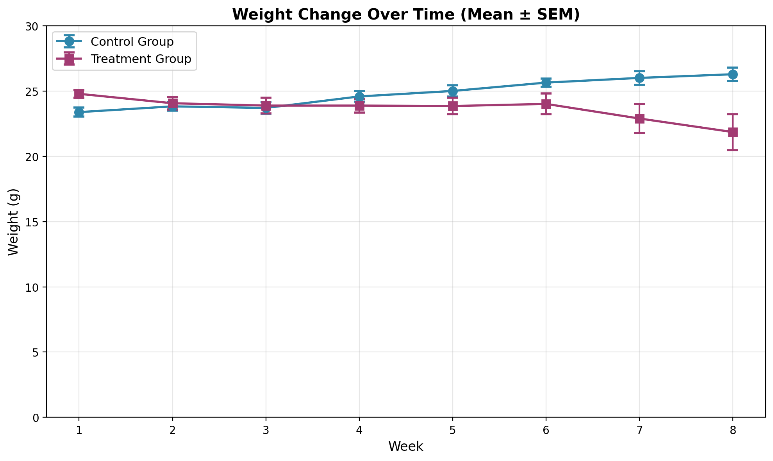


**Figure S1** Weight changes in control and treatment groups over an 8-week period. Mice were weighed weekly and data are presented as mean ± SEM (n=5 per group). Control mice (blue circles, solid line) showed steady weight gain throughout the study period, increasing from 23.4 g at week 1 to 26.3 g at week 8. Treatment mice (purple squares, dashed line) exhibited weight loss over time, decreasing from 24.8 g at week 1 to 21.9 g at week 8, demonstrating a significant divergence from the control group.


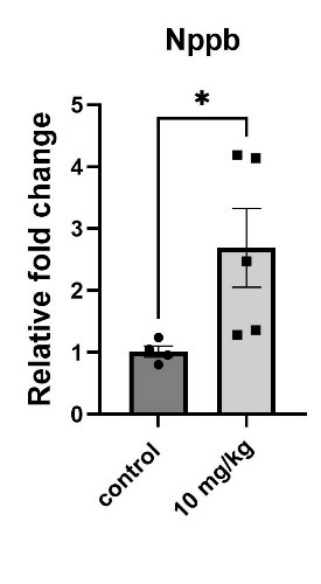

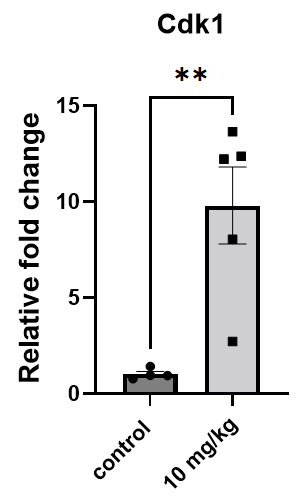

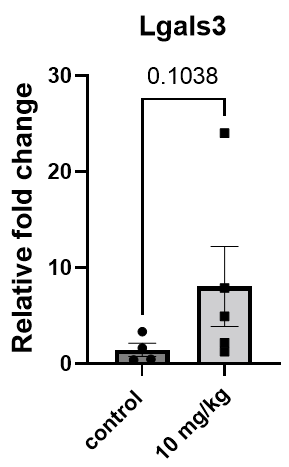


**Figure S2** Changes in expression of markers of heart damage in mice by qPCR-RT in oxaliplatin (10 mg/kg, 8 dosages weekly) vs vehicle (control). * *p* < 0.05, ** *p* <0.01, others not significant. N= 5 per group


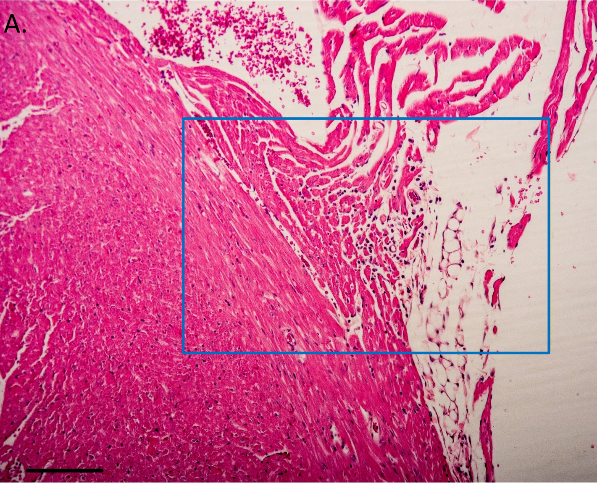

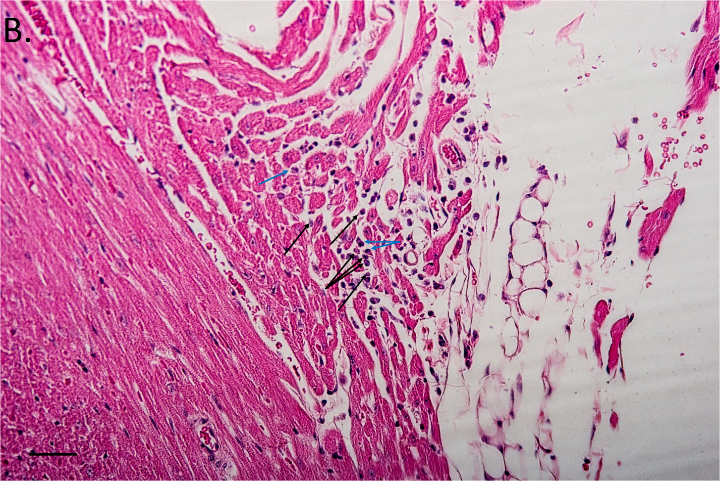


**Supplementary Figure S3**. Photomicrograph of histology of section near right atrium. **A)** Low magnification image from near right atrium. Area surrounded by the blue box is shown in B. Scale bar = 300 µm. **B)** Higher magnification image from A. Interstitial infiltration by neutrophils (black arrows) and mononuclear cells (blue arrows). Scale bar = 100 µm.


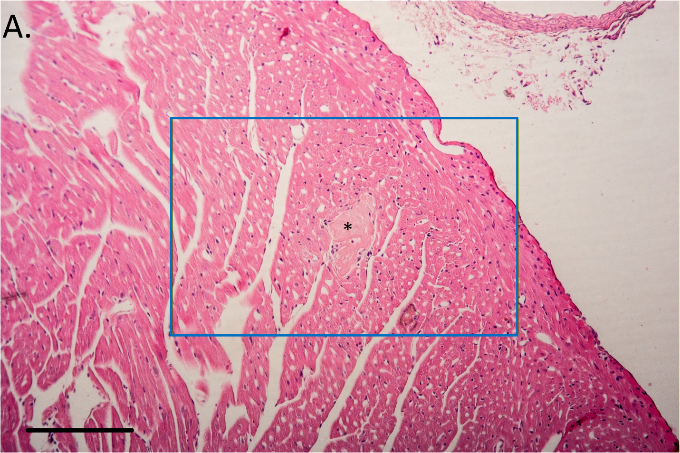

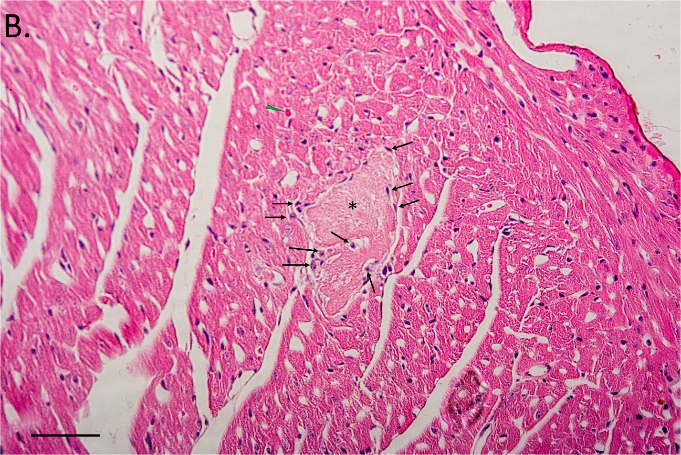


**Supplementary Figure S4**. Photomicrograph of histology of the mouse cardiac apex. **A)** Low magnification image from near the apex of the mouse heart with focal necrosis (*). Area surrounded by the blue box is shown in B. Scale bar = 300 µm. **B)** Higher magnification image from A. Focal necrosis (*) with numerous neutrophils (black arrows) and an eosinophil (green arrowhead) present in B. Scale bar = 100 µm.


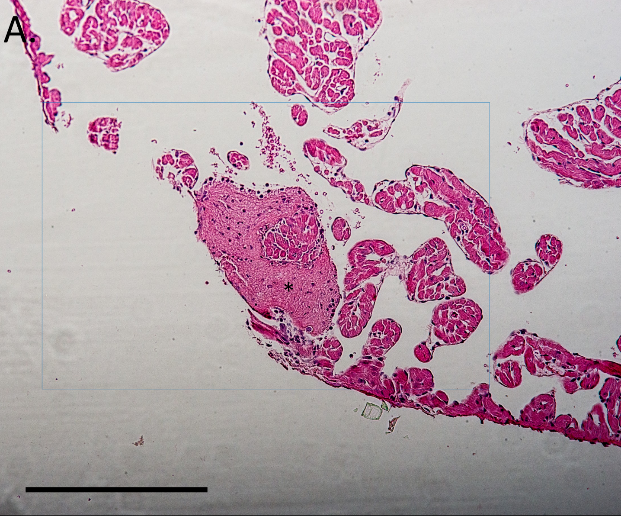

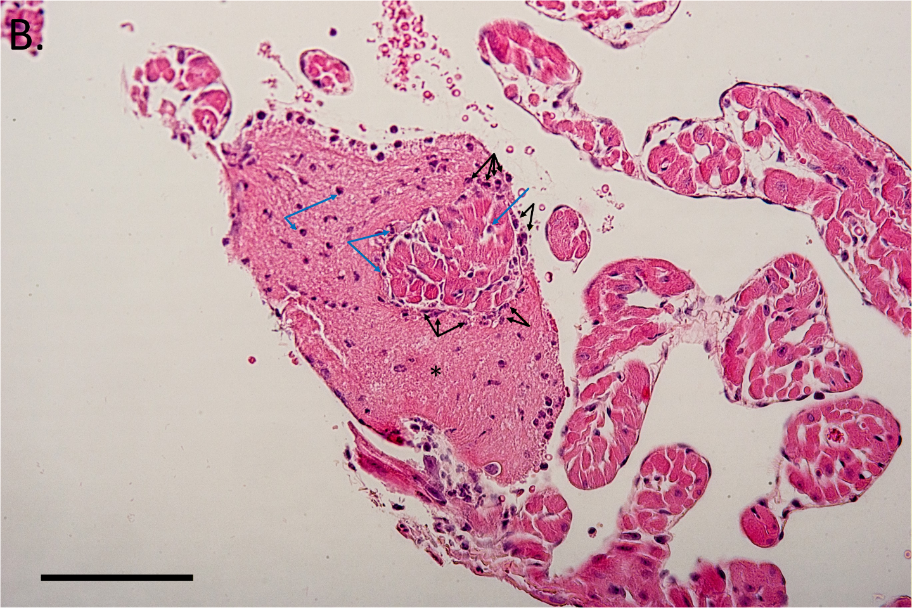


**Supplementary Figure S5**. Photomicrograph of histology of the mouse right atria. **A)** Low magnification image from near the right atrial appendage with focal necrosis (*). Area surrounded by the blue box is shown in B. Scale bar = 300 µm. **B)** Higher magnification image from A. Focal myocardial necrosis (*) is present within the with numerous neutrophils (black arrows) and mononuclear cells (blue arrows) in the right atrial appendage. Scale bar = 100 µm.


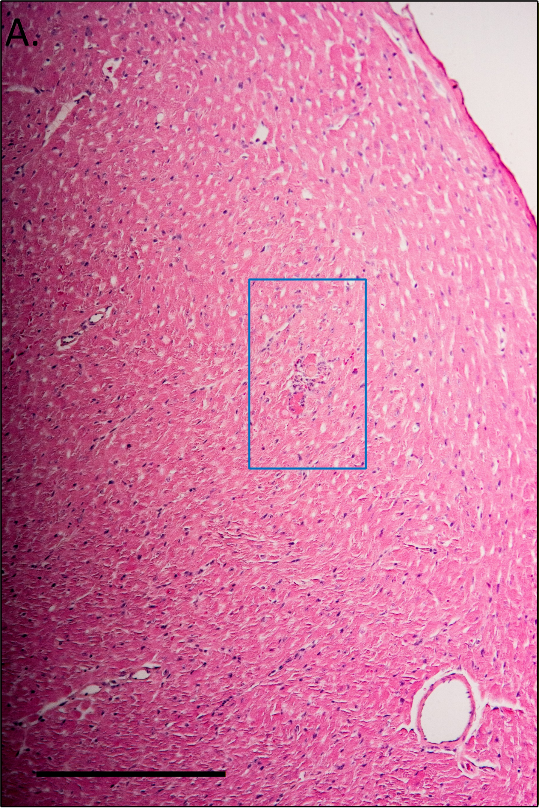

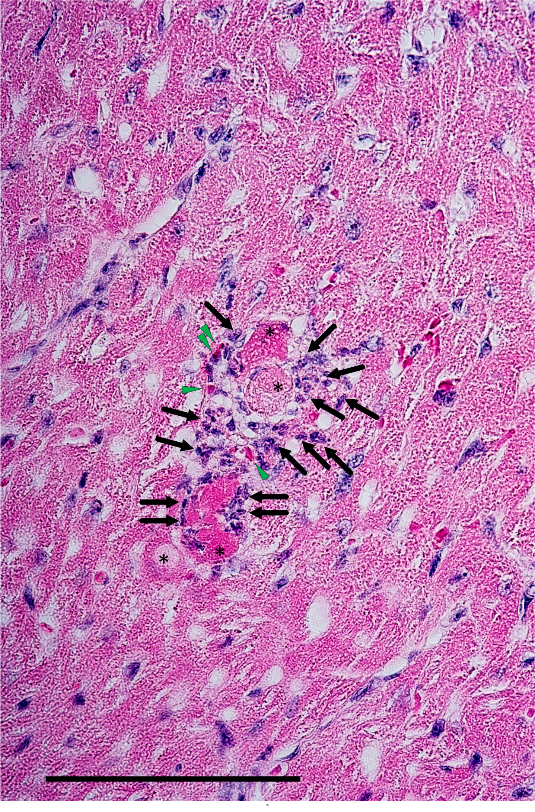


**Supplementary Figure S6**. Photomicrograph of histology in the mouse left ventricle. **A)** Low magnification image from left ventricular myocardium. Area surrounded by the blue box is shown in B. Scale bar = 300 µm. **B)** Higher magnification of image from A. There are a few minimal foci of cardiomyocyte necrosis (*) characterized by fiber hypereosinophilia (green arrowheads), fragmentation, and neutrophil infiltration (black arrows). In some areas there appears to be mild freeze artifacts, with interstitial vacuolation and infrequent cytoplasmic granularity. Scale bar = 100 µm.


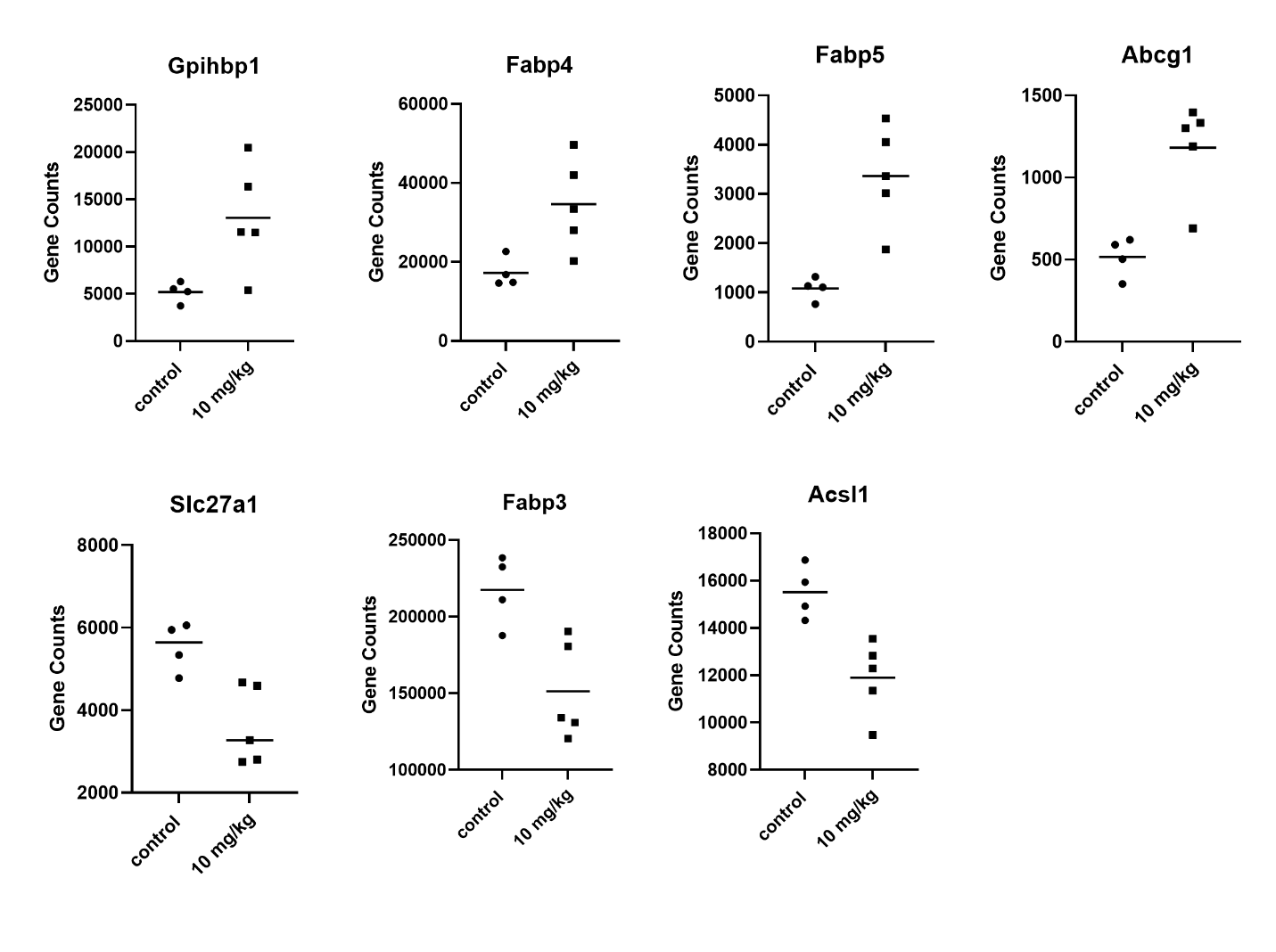


**Figure S3** **7** Oxaliplatin upregulates genes responsible for transport of FAs from capillary lumen to endothelium: *Gpihbp1* (FC =2.51, FDR=8.56E-5), *Fabp4* (FC = 2.01, FDR=2.54E-4) and *Fabp5 (*FC= 3.12, FDR=2.79E-10). Oxaliplatin downregulates genes responsible for transport of FAs from endothelium to mitochondrion of cardiomyocytes: *Fabp3* (FC = −1.44, FDR=9.45E-3), *Acsl1* (FC = −1.3, FDR=0.02). Based on RNA-seq analysis on *n* = 4 control and *n* = 5 oxaliplatin-treated mice.


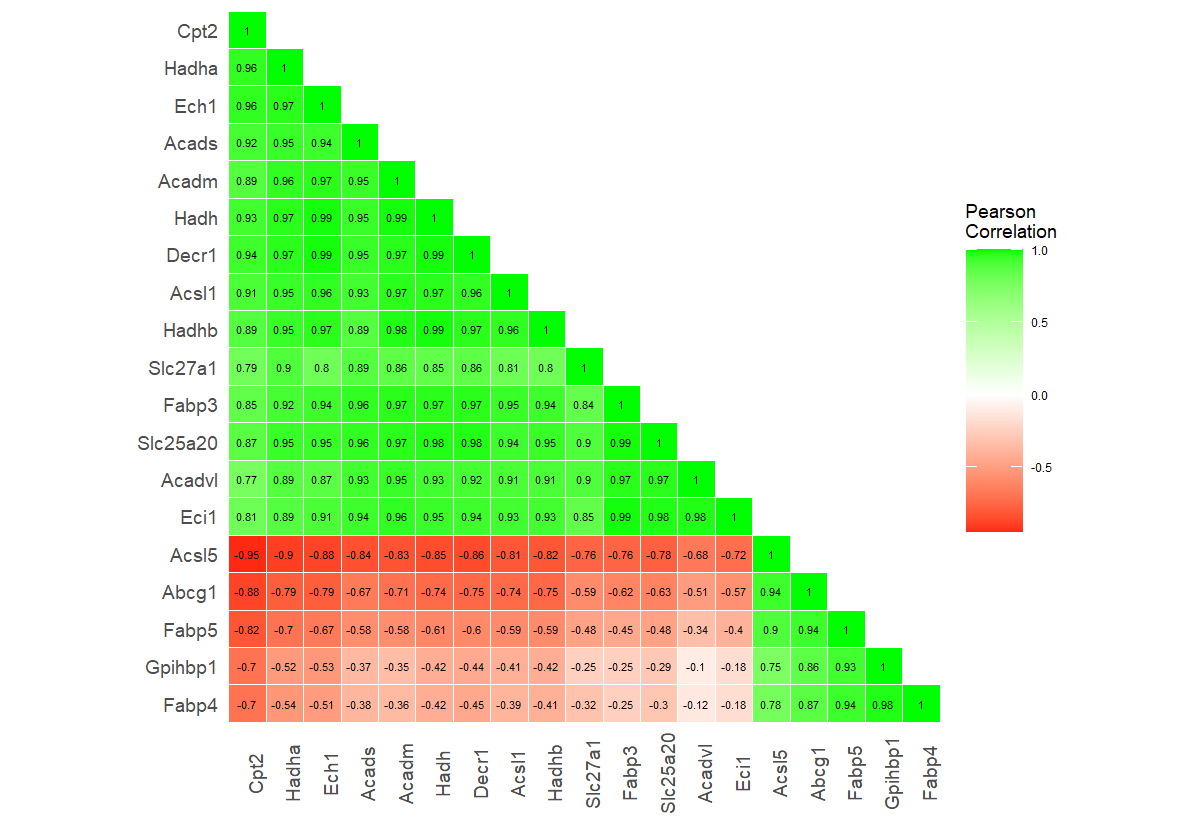


**Figure S****8** Correlation matrix of differentially expressed genes: FA transport and oxidation process:


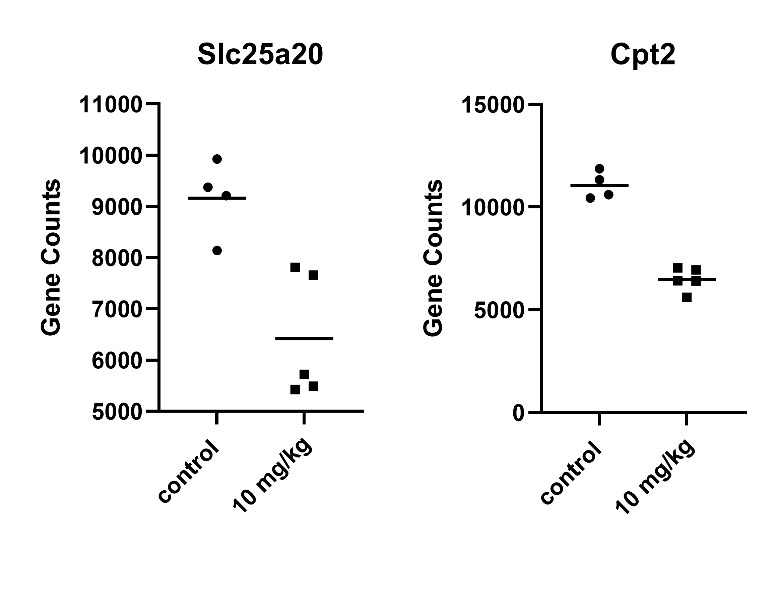


**Figure S9** Key proteins in the FA transport through mitochondria membrane are downregulated: *Slc25a20* (FC= −1.43, FDR = 4.99E-3) and *Cpt2* (FC = −1.71, FDR = 8.6E-12). Based on RNA-seq analysis on *n* = 4 control and *n* = 5 oxaliplatin-treated mice.

**Figure S****10** Effect of oxaliplatin on β-oxidation of saturated FAs. Oxaliplatin downregulates three of the four Acyl-CoA dehydrogenases and a thiolase: short chain FA (SCAD, *Acads* (FC = −1.47, FDR=4.55E-4), medium chain FA *Acadm* (FC = −1.43, FDR=2.23E-3) and very long chain FA *Acadvl* (FC = −1.31, FDR=0.05), that corresponds to short, medium, and very long FAs. Dehydrogenation enzymes and thiolysis enzyme are also downregulated: NADH, encoded by *Nadh* (FC= −1.5, FDR = 2.93E-4), *Nadha* (FC= −1.53, FDR = 2.5E-6), *Nadhb* (FC *=* −1.47, FDR 1.9E-3). Based on RNA-seq analysis on *n* = 4 control and *n* = 5 oxaliplatin-treated mice.


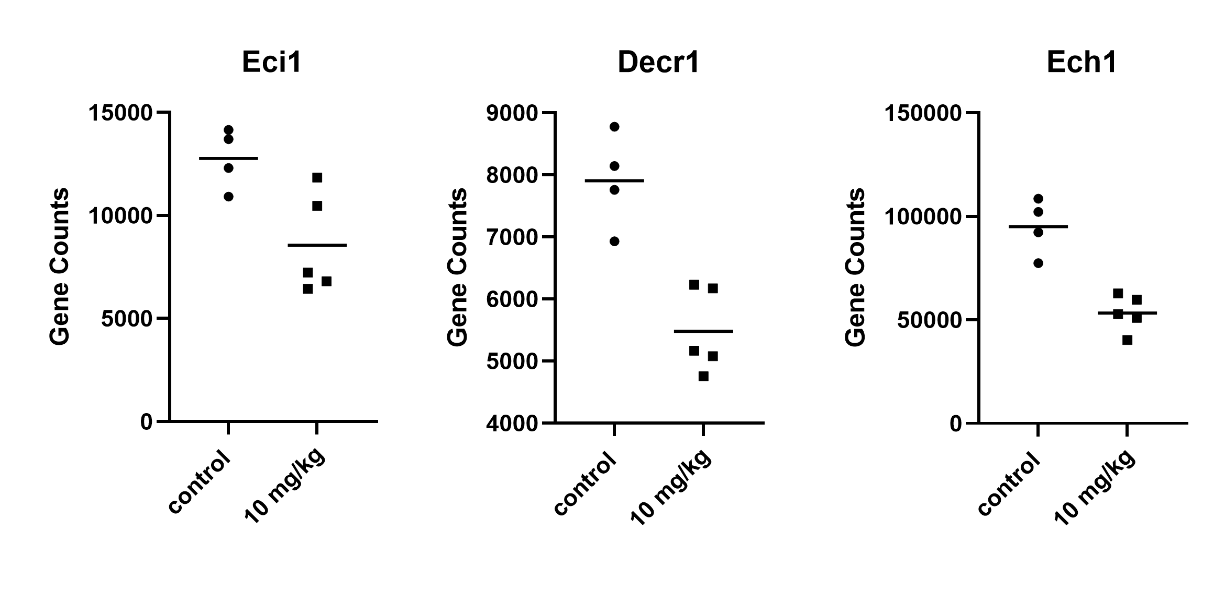


**Figure S****11** Effect of oxaliplatin on β-oxidation of unsaturated FAs results on downregulation of several key genes: *Eci1* (FC = −1.49, FDR = 0.02); *Decr1* (FC = −1.44, FDR = 3.49E-4), *Ech1* (FC = −1.78, 1.89E-6) Based on RNA-seq analysis on *n* = 4 control and *n* = 5 oxaliplatin-treated mice.


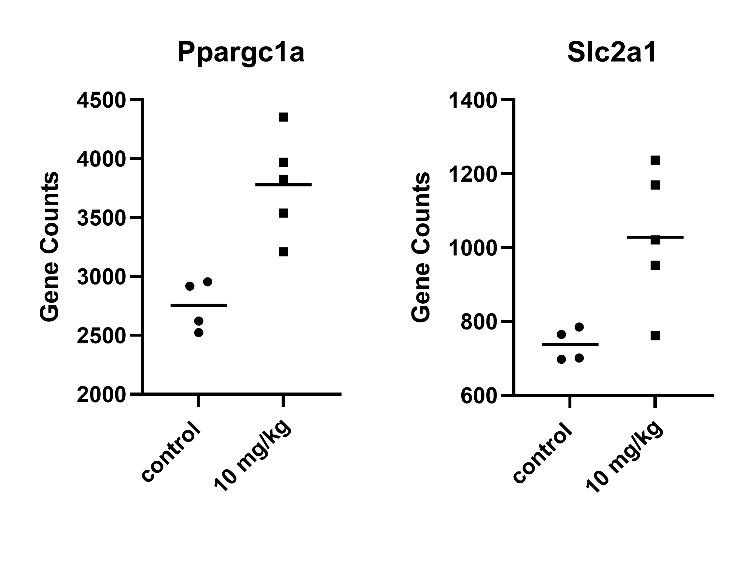


**Figure S12** Oxaliplatin upregulates the energy-sensing enzyme *Ppargc1a* (FC=1.37-fold increase, FDR = 1.39E-3). Increase of glucose transport by *Slc2a1* (GLUT1) (FC = 1.39-fold increase, FDR = 0.01) suggests a partial switch to glycolysis. Based on RNA-seq analysis on *n* = 4 control and *n* = 5 oxaliplatin-treated mice.


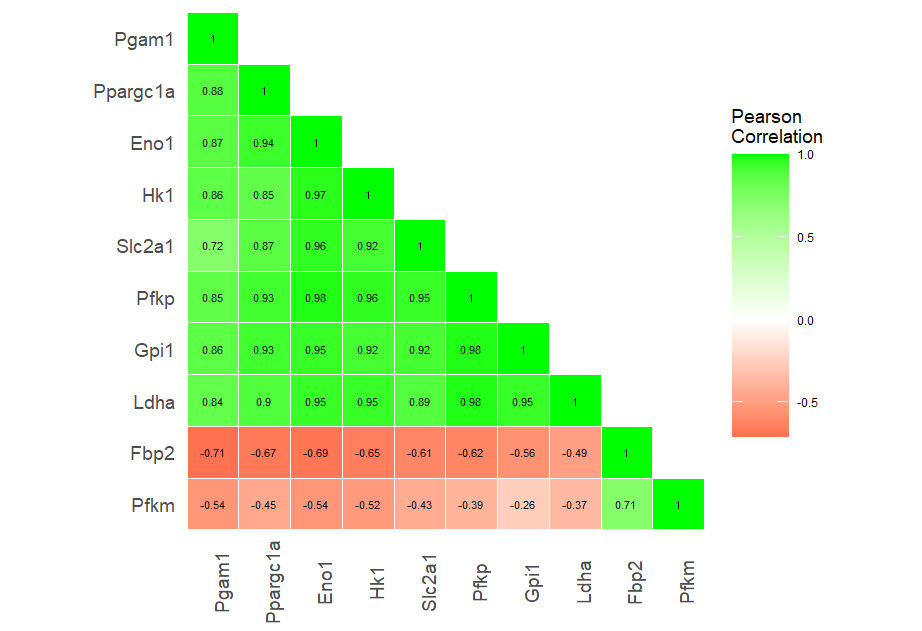


**Figure S13** Correlation matrix of differentially expressed genes. Glycolysis pathway. Based on RNA-seq analysis on *n* = 4 control and *n* = 5 oxaliplatin-treated mice.


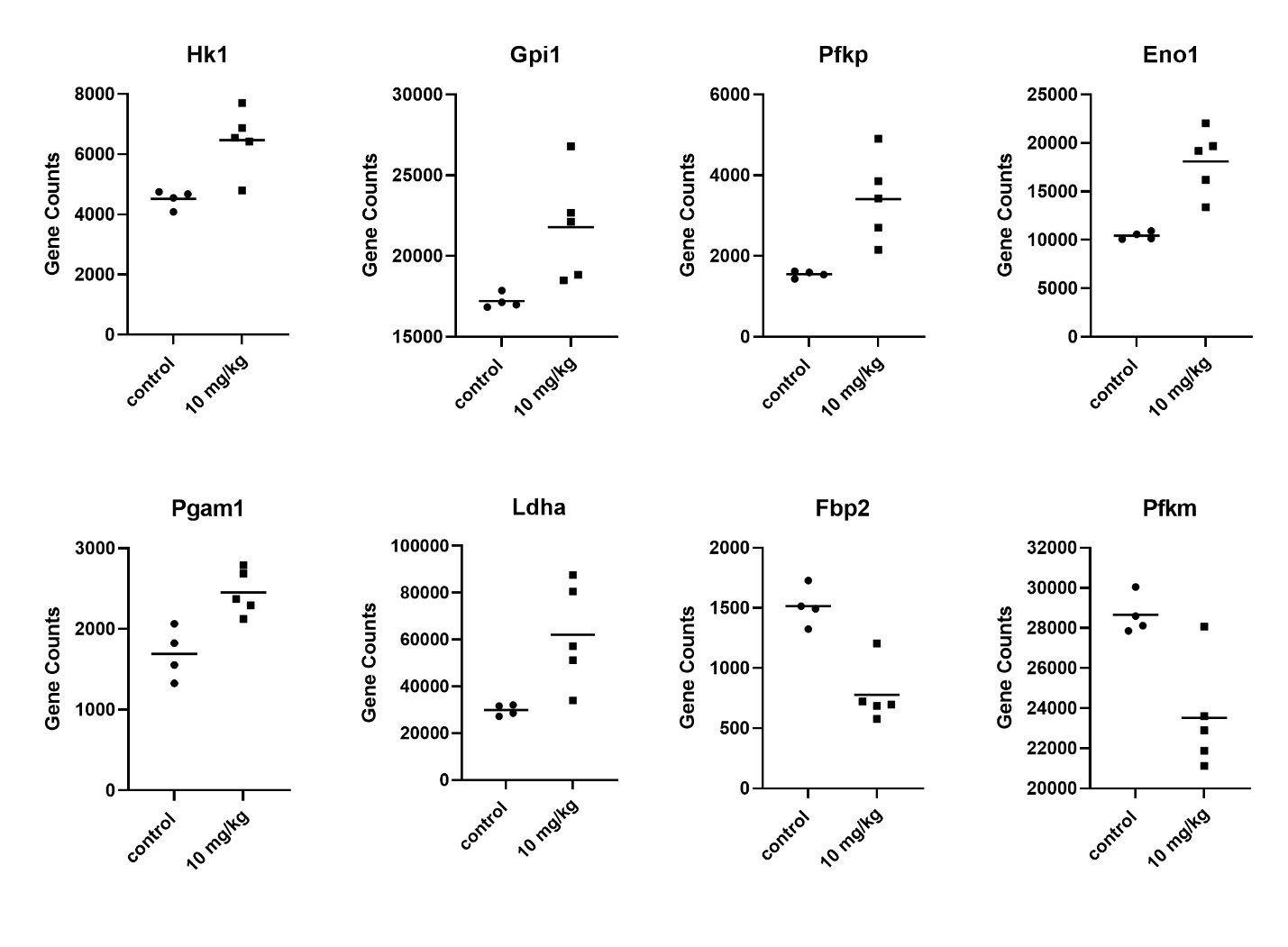


**Figure S14** Oxaliplatin leads to significant changes in gene expression in glycolysis pathway. Upregulated: *Hk1* (FC = 1.43, FDR = 2.29E-3), *Gpi1* (FC = 1.27, FDR = 0.04, *Pfkp* (FC = 2.2, FDR = 1.2E-6), *Eno1* (FC = 1.74, FDR = 1.84E-6), *Pgam1* (FC = 1.45, FDR = 3.97E-3), *Ldha* (FC = 2.08, FDR = 7.89E-05); *Fbp2* (FC = −1.95, FDR = 4.18e-5) and *Pfkm* (FC = −1.22, FDR = 0.04) are downregulated. Based on RNA-seq analysis on *n* = 4 control and *n* = 5 oxaliplatin-treated mice.


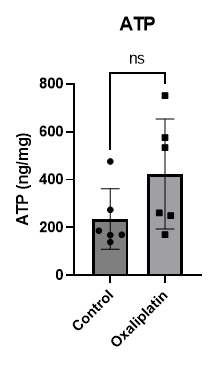


**Figure S15** The level of ATP in the heart is not statistically significantly changed indicating an ATP compensation from glycolysis
